# A gestural repertoire of 1- to 2-year-old human children: in search of the ape gestures

**DOI:** 10.1007/s10071-018-1213-z

**Published:** 2018-09-08

**Authors:** Verena Kersken, Juan-Carlos Gómez, Ulf Liszkowski, Adrian Soldati, Catherine Hobaiter

**Affiliations:** 1 School of Psychology and Neuroscience, University of St Andrews, St Andrews, KY16 9JP Scotland UK; 2 Budongo Conservation Field Station, P.O. Box 32, Masindi, Uganda; 3 Department of Cognitive Developmental Psychology, University of Göttingen, Waldweg 26, 37073 Göttingen, Germany; 4 Department of Developmental Psychology, Hamburg University, Von-Melle-Park 5, 20146 Hamburg, Germany; 5 Department of Comparative Cognition, University of Neuchatel, Neuchatel, Switzerland

**Keywords:** Children, Gesture, Chimpanzee, Language, Reference

## Abstract

When we compare human gestures to those of other apes, it looks at first like there is nothing much to compare at all. In adult humans, gestures are thought to be a window into the thought processes accompanying language, and sign languages are equal to spoken language with all of its features. Some research firmly emphasises the differences between human gestures and those of other apes; however, the question about whether there are any commonalities is rarely investigated, and has mostly been confined to pointing gestures. The gestural repertoires of nonhuman ape species have been carefully studied and described with regard to their form and function—but similar approaches are much rarer in the study of human gestures. This paper applies the methodology commonly used in the study of nonhuman ape gestures to the gestural communication of human children in their second year of life. We recorded (*n* = 13) children’s gestures in a natural setting with peers and caregivers in Germany and Uganda. Children employed 52 distinct gestures, 46 (89%) of which are present in the chimpanzee repertoire. Like chimpanzees, they used them both singly, and in sequences, and employed individual gestures flexibly towards different goals.

## Introduction

In this paper we make a first attempt at classifying the natural gestural repertoire of human infants in their second year of life with the same methodology that has been applied to other ape species. We aim to show that applying this methodology to the study of human infant gesture is not only a worthwhile endeavour that can tell us more about the development of human communication, but also supplement what we know about the similarities and differences to the communication systems of other apes.

Great apes of all species—human and nonhuman—communicate using a combination of different types of signals: vocalizations, gestures, facial expressions, body postures, and even cues from colour, such as blushing (deJong 1999), or odour (Singh and Bronstad 2001; Hepper and Wells 2010) can transmit information between individuals. In humans, however, language (whether spoken or signed) appears to represent a fundamentally distinct system of communication, with its flexible production and recursive properties allowing extraordinary potential for the expression of a near-infinite range of meanings, intentionally addressed towards highly specific audiences.

Humans also produce non-intentional signals—the yelp when we stub our toe clearly conveys the information that we are in pain to anyone else in the room, but the signaller did not yelp with an *intention* to communicate this information. Early studies of human communicative development distinguished intentional (illocutory) signals, from nonintentional (perlocutory) signals, in which a signal may have an effect on a recipient but without any evidence that the signaller intended this effect (Bates et al. 1975). Unlike human language, nonhuman vocal communication largely fits into this second—nonintentional category (Cheney and Seyfarth 1990; Marler 1961; Seyfarth and Cheney 2002). One exception is the evidence for the intentional use of a specific alarm call by chimpanzees (Crockford et al. 2012, 2017; Schel et al. 2013); however, this remains limited to an individual signal used in a highly specified way. Great ape gesture is different. Non-human great apes (hereafter great apes) have large species repertoires of over 60 different gesture types (Hobaiter and Byrne 2011a, b; Byrne et al. 2017), with widespread evidence across all species that apes deploy these both flexibly and intentionally (e.g. Tomasello et al. 1985; Tanner and Byrne 1993; Pika et al. 2005; Liebal et al. 2006; Genty et al. 2009; Cartmill and Byrne 2010; Hobaiter and Byrne 2011a, b; Roberts et al. 2012; Frohlich et al. 2016). Current studies suggest that great ape gesture is the only nonhuman *system* of communication in which, like language, a repertoire of signals is used intentionally to communicate everyday goals. As a result, it has been argued that human language may have originated in the gestural domain (Hewes et al. 1973; Armstrong et al. 1994, 1995; Corballis 2002, 2003; Tomasello 2008).

Recent comparative studies across great ape species have revealed that there is substantial overlap in both the repertoires of available gesture types (Hobaiter and Byrne 2011a, b, and see; Byrne et al. 2017), and, in some cases, in the meanings for which great apes use these gestures (Graham et al. 2018). As a result, it has been suggested that there is a large ‘ape-typical’ repertoire of gesture types (Hobaiter and Byrne 2011a, b; Cartmill et al. 2011, and see; Byrne et al. 2017). Large similarities in the gestural repertoire have been observed in apes with very different ecologies, social structures, and cognitive skills (Pika et al. 2005)—if the gestural repertoire is shared across modern ape species despite these variations, this strengthens the hypothesis that modern ape gestural repertoires originated in a shared universal ancestral repertoire. A biological shared repertoire of available gesture types presents an interesting question. Chimpanzees and bonobos are much more closely related to humans, than they are to gorillas or orang-utans (Langergraber et al. 2012). At least 36 gesture types are shared amongst all of the nonhuman apes species, with even larger overlaps between gorillas and chimpanzees or bonobos (Byrne et al. 2017). So what happened to this repertoire in humans? It is an open question whether any of these ape gestures are also present in the human repertoire before language typically becomes the main means of communication, and, if present, whether they persist afterwards alongside language. Given this biological puzzle, a recent paper posed the question: ‘Where have all the (ape) gestures gone?’ (Byrne and Cochet 2016)—have they disappeared with changing evolutionary-developmental constraints in the course of human evolution, or have they been missed in research on early human communication?

Part of the reason why this question remains open, despite an extensive literature comparing communication of humans and other apes (e.g. Premack 1971; Premack and Premack 1972; Hewes et al. 1973; Miles 1983; Savage-Rumbaugh 1988; Deacon 1989; Snowdon 1990; Corballis 1992; Savage-Rumbaugh et al. 1993; Povinelli et al. 1997; Tomasello and Camaioni 1997; Burling et al. 1993; Fitch 2000, 2005; Tomasello and Zuberbühler 2002; Brinck and Gärdenfors 2003; Dunbar 2003; Gomez 2007; Pollick and DeWaal 2007; Tomasello 2007, 2010; Seyfarth and Cheney 2008; Liszkowski et al. 2009; Fedurek and Slocombe 2011; Gibson 2012; Scott-Phillips et al. 2015; Moore 2016; Zuberbühler and Gómez 2018), is that studies on human and nonhuman apes rely on different methodologies and definitions, and conceptualizations of gesture and communication (Scott and Pika 2012; Gillespie-Lynch et al. 2014; Fröhlich and Hobaiter 2018). For example, in adult humans, a major research goal is to investigate how gesture contributes to the acquisition and use of speech and the interaction between gestures and thought (e.g. Kendon 2004; Goldin-Meadow 1999; Goldin-Meadow and Alibali 2013). Thus, many human developmental studies focus on the development of referential and deictic gestures, such as pointing, and their connection with language acquisition and intentional reference. This, in turn, has influenced primate studies that have frequently addressed the issue of to what extent apes have similar referential gestures and to what extent they are used with communicative intent. Indeed, given the claims that the capacity for intentional communication was uniquely human (e.g. Scott-Phillips 2010, 2015, 2016), a major focus of much gestural research in nonhuman primates was its intentional use. Most comparative nonverbal communicative research that incorporates both human and nonhuman primates has focused on facial and vocal expressions (e.g. Matsumoto et al. 2016), but virtually no study has tried to directly compare the gestural *repertoires* of humans and other apes.

As very young children do not yet rely on spoken or signed language as their primary means of communication, they may be better suited for a comparison with nonhuman primate communication. Like other great apes, we need to rely on observable behaviour to interpret and classify their communicative attempts. Gesture has played a prominent role in studies of early childhood language acquisition, with an emphasis on gestures as the beginning of intentional communication and as tools that help children in language acquisition (e.g. Bruner 1981, Bates et al. 1975, 1979). A central question of gestural research with prelinguistic infants is whether features of language (symbolicity, reference) are present even before the onset of spoken or signed language. Infant gestures have been roughly divided into two categories: imperative gestures (mainly requests) and declarative gestures (gestures with the aim of directing another’s attention to some aspect of the environment for the sake of sharing attention). Most attention has been given to the class of declarative gestures since these are more similar to specifically human forms of communication (e.g. Murphy 1978; Leung and Rheingold 1981; Bates et al. 1989; Franco and Butterworth 1991, 1996; Povinelli et al. 1997; Carpenter et al. 1998; Kita 2003a, b; Butterworth 2003; Legerstee and Barillas 2003; Camaioni et al. 2004; Goldin-Meadow 2007; Liszkowski et al. 2004; Southgate et al. 2007; Tomasello et al. 2007; Tomasello 2008; Liszkowski 2008, 2010; Behne et al. 2012). Deictic gestures are culturally universal (e.g. Blake et al. 2005, Liszkowski 2005; Liszkowski et al. 2012; Salomo and Liszkowski 2013) and appear in both deaf children who have no language input from signing parents (Goldin-Meadow and Feldman 1977, 1975; Caselli 1983; Acredolo and Goodwyn 1990; Goldin-Meadow 1993, 2002, 2005; Spencer 1993; Iverson et al. 1994; Pien 1984; Robinshaw 1996; Lederberg and Everhart 1998; Volterra et al. 2006) and blind children who do not get visual input (Moore and McConachie 1994; Preisler 1995; Iverson and Goldin-Meadow 1997; Iverson et al. 2000; Bruce et al. 2007). The use of deictic gestures predicts language onset and vocabulary in later development, cementing the link between prelinguisitc gesture and language (e.g. Dobrich and Scarborough 1984; Bates et al. 1989; Caselli 1990; Harris et al. 1995; Butterworth and Morisette 1996, Tomasello and Camaioni 1997; Goodwyn and Acredolo 1998; Iverson et al. 1998; Goodwyn et al. 2000; Capirci et al. 2002, 2005; Kita 2003a, b; Iverson and Goldin-Meadow 2005; Özçalışkan and Goldin-Meadow 2005; Volterra et al. 2005; Bavin et al. 2008; Capirci and Volterra 2008; Kelly et al. 2008; Rowe et al. 2008; Rowe and Goldin-Meadow 2009; Gliga and Csibra 2009; Colonnesi et al. 2010). As children learn their first words they combine these with their existing gestures, paving the way for the later development of multi-word combinations (for a review see Goldin-Meadow and Alibali 2013, for other examples see:; Morford and Goldin-Meadow 1992; Capirci et al. 1996; Iverson and Thelen 1999; Butcher 2000).

One gesture that has been frequently compared and contrasted in human infants and other apes is index finger pointing. In human infants, pointing reliably emerges at around 9 months of age, shows intentionality and flexibility, and seems to be universal across cultures (Kita 2003a, b, 2009; Liszkowski 2005), although with differences in form (Kita 2003a, b). Human infants use pointing in many different, triadic contexts: to request an action or object, to share interest and attitudes about something, to helpfully provide information for a recipient (Tomasello et al. 2007; Liszkowski et al. 2007; Liszkowski 2005; Liszkowski et al. 2006), and even to ask for more information (Southgate et al. 2007). In other apes, however, index finger pointing appears absent in wild populations, and is typically limited to a requesting function in captive apes (Gómez et al. 1993; Call and Tomasello 1996; Krause 1997; Tomasello and Camaioni 1997; Tomasello 2007, 2008; Bullinger et al. 2011) who also tend to produce a whole-hand rather than index-finger version of pointing (e.g. Leavens and Hopkins 1999). The performance of apes and human children has been directly compared in a number of laboratory settings, finding remarkable differences between the ape species (e.g. Povinelli et al. 1997; Liszkowski et al. 2009; Goot et al. 2014; but c.f.; Leavens et al. 2017). To date, it seems reasonable to conclude that index finger pointing, particularly in declarative contexts, is either absent or rare in the natural communication of other apes (althought c.f Leavens et al. 2005, 2017; and Lyn et al. 2010). But what about other gestures in the infant’s repertoire?

Early studies of infant gestures used a more ethological approach to describe the gestural repertoire of human children in various settings: they observed infants in natural interactions with their caregivers, recorded the form and frequency of the gestures, and took into account the receiver’s reaction to these gestures (for example Blake et al. 1992; Morris 1994; Iverson and Goldin-Meadow 2005; Acredolo and Gooodwyn 1985; Caselli 1983; Volterra 1981). These studies were, however, largely subordinated to the study of language acquisition, and how gesturing influences and enhances this process. For example, Acredolo and Goodwyn (1988) asked mothers of 38 children about their infants’ gestures. As part of a diary study, mothers gave information about the form, frequency, and context in which the gestures were observed. Acredolo and Goodwyn (1988) reported 148 gestures that occurred frequently in several contexts: as object gestures that referred to a specific object or thing, as requests, as attributes about certain objects, as replies to questions or suggestions from interactive partners, and lastly as part of specific events, for example waving ‘bye-bye’. Similarly, Blake et al. (1992) used video observations to map the gestural repertoire of 10 children in their second year of life. Interestingly, in addition to deictic gestures (comment gestures, referring to objects or as part of ritualized displays), she also reported emotive, protest, and request gestures that she speculated could be similar to those of other apes. In a longitudinal, cross-cultural observation, Blake et al. found that these imperative classes of gestures declined and the classes of deictic gestures increased, as children got older and their spoken vocabulary increased (Blake et al. 2005). The same developmental pattern was reported in children from all cultures included in the study: Japan, Franco-Canadian, Italian-Canadian and French. From these longitudinal observations of children in their natural social environment, Blake developed descriptions of their gestures and found that at least some of these could be compared to ape gestures (Blake 2000). Similarly, Gillespie-Lynch et al. (2014) directly compared gestures of language-trained chimpanzees, bonobos, and human children. Using the same methodology and definition of gestures, they reported striking similarities in some gestures across human and nonhuman apes; however, as in previous studies, nonhuman ape species performed few deictic gestures and these did not increase with age. In contrast, Tomasello and Camaioni (1997), found little similarity between children and ape gestures, and they suggest that the gestural repertoire of human children differs from infancy with regard to form, function, and underlying motif (see also Call and Tomasello 2007).

A final series of studies allowing some comparison is seen in the work of Golinkoff (human infants: 1986); Leavens et al. (chimpanzees: 2005a) and Cartmill and Byrne (orang-utans: 2007). Here, an infant, chimpanzee, or orang-utan was shown a desirable object and requested this by gesturing. The communicative partner then complied with their request, partially met it, or misunderstood it completely. Independently of the particular gestures used, both children and apes showed the same general pattern in their gestural behaviour: They stopped gesturing when the request was met, elaborated when it was partially met, and used different gestures when they were misunderstood. Some of the gesture types, for example: ‘pointing’, ‘banging’, and ‘reaching’ were recorded in both children and apes.

In sum, we do not currently have a comprehensive description of the gestural repertoire of 1- to 2-year-old human infants. Textbook descriptions (e.g., Lock and Zukoff-Golding 2010) tend to discuss child gestures in the second year of life in terms of deictic gestures (e.g., pointing with different forms and functions, reaching, showing and offering objects) complemented by conventional gestures specific to each culture (‘bye, bye’), and later developing iconic or symbolic gestures like twisting movements of a hand to request opening a jar (Lock and Zukoff-Golding 2010; Pika 2008). There is reference to the existence of other, largely unspecified “idiosyncratic” gestures in individual infants displayed as “excerpts of direct actions” to make requests (Lock and Zukoff-Golding 2010), and indeed in many individual studies we find occasional descriptions of non-deictic gestures. For example, raising arms to request being picked up (Lock 1980) or hand-leading to request displacements (Gómez 2015), but no systematic description of the general repertoire of gestures of children.

Although a review of the literature shows some suggestive evidence that at least a part of human infants’ gestures may be shared with other primates, very few studies observe gestures from human infants and other primates in their natural environment and classify these using the same methodological approach (Pika 2008; Gillespie-Lynch et al. 2014). In order to address the question of whether human infants share in an ape-typical repertoire, ideally, we would employ the same method of data collection and definition and description of gesture for all species concerned. This approach does not imply fitting a square peg into a round hole: human and nonhuman apes are distinct in their ecology and sociality. Ethological methods consider these aspects and can provide us with a full picture of gestural communication, including both shared and unique forms.

The best approach to generate data suitable for a comparison is to employ a methodology that is already widely used across nonhuman great ape species (e.g. Tomasello et al. 1985; Tanner and Byrne 1993; Pika et al. 2005; Liebal et al. 2006; Genty et al. 2009; Cartmill and Byrne 2010; Hobaiter and Byrne 2011a, b; Roberts et al. 2012; Frohlich et al. 2016). This methodological approach was initially piloted by Ladygina-Kohts (1935) in her comparison of expressive behaviour of her own child and a juvenile chimpanzee, and further developed in the 1960s (e.g. Van Hoof 1967; Van Lawick-Goodall 1972; Plooij 1978). In this approach any possible action of the body can be considered a gesture, so long as it is accompanied by clear behavioural indications of intentional use. Typically criteria for ascribing intentional use are based on those employed by Bates et al. (1975) for use with preverbal human infants: an awareness of the recipient and their attentional state, waiting for the recipient to respond after performing a gesture, evaluating this response and then acting accordingly: persisting when the recipient did not react, stopping to signal when a satisfactory outcome has been achieved, and changing strategy when an undesired outcome has been achieved (e.g. Tomasello et al. 1985; Tanner and Byrne 1993; Pika et al. 2005; Leavens and Hopkins 1998; Leavens et al. 2005a, b; Liebal et al. 2006; Genty et al. 2009; Cartmill and Byrne 2010; Hobaiter and Byrne 2011a, b; Roberts et al. 2012; Frohlich et al. 2016). By adopting this methodology we are able to use behavioural criteria of intentionality in the sense of goal directedness, without implying particular cognitive processes of communicative intent or mental state attribution.

This paper is a first attempt to compare the repertoire of gestures of human infants to those of other apes using a methodological approach that is directly comparable. Our results report both the types of gestures and the frequency in which we observed them in everyday interactions.

Following previous studies, our subjects were human infants in their second year of life whom we observed in their natural habitats at home and at a daytime nursery. Previous studies have shown that gesturing is influenced by children’s native language (Kelly et al. 2008; Kita et al. 2007; Pika et al. 2006; Kita 2003a, b; Capirci et al. 1998) and by specific cultural interaction pattern (Salomo and Liszkowski 2013; Kita 2003a, b, 2009). Our sample population came from two different cultures, German and Ugandan, to reduce bias from the impact of culture and native language on early gesturing. Both cultures differ in terms of parental care and social interaction with children. German children make up a typical WEIRD sample (Henrich et al. 2010a, b; Hwang 2013; Keller and Kärtner 2013; Henrich 2015), in comparison Ugandan children grow up in larger family groups and often have siblings and other young relatives as primary contacts. Furthermore, studies observed lower rates of triadic interaction between the child, a parent or caregiver, and an object or event in the environment in Ugandan children (Kersken et al. 2017, see also; Britto et al. 2013; Salomo and Liszkowski 2013; Kärtner et al. 2010; Keller et al. 2005; Rabain-Jamin et al. 2003; Ainsworth 1967). Given the small sample from our two populations our goal is not to engage in cross-cultural comparison; instead we incorporate both populations to provide a more culturally diverse dataset in order to better start to describe any species-typical human behaviour.

## Methods

In this study, we analysed children’s gestures in video recordings of human infants during their natural interaction with others (peers, caregivers, relatives) either in their home compound (Uganda) or in a daytime nursery (Germany). Human communication involves biologically inherited species-wide traits, such as a shared repertoire of potentially available phonemes in early infancy (Lenneberg 1968), or the emergence of hierarchically structured grammar (Sandler et al. 2005; Senghas et al. 2004; Senghas and Coppola 2001). At the same time, there is a strong impact of social learning—leading to the many languages, dialects, and cultural norms (Pinker 2003; Gregory and Carroll 1978; Halliday 1975). Language itself interacts with other culturally specific behaviour, such as teaching or social learning (Schieffelin and Ochs 1986; Howard et al. 2014; Shneidmann et al. 2016). Our aim here was to demonstrate the validity of applying the nonhuman ape method to human children, to explore any possible use of the great-ape repertoire of gestures. In order to reduce bias created by culturally- or environmentally-specific features of a specific human population, as well as collecting data specifically for this study (Göttingen, Germany), we incorporated opportunistic coding of an existing data collected as part of an observational study investigating children’s everyday interactions with their caregivers in Masindi, Uganda (see Kersken et al. 2017).

### Populations studied

Uganda: The spontaneously occurring behaviour of seven children (four girls and three boys) aged 315–421 days, was observed and recorded in their home environment. The children’s mother was always present or nearby and children were free to move around their home and interact with their family members (siblings, cousins, parents, and grandparents for example), or with other children of either the same age or older. Children were observed on and around their family’s compound that was often shared with extended family. Compounds consisted of several simple houses with no access electricity or running water. Animals like chickens or goats were often housed on these compounds. Parents were subsistence farmers or, mainly in case of the fathers, had jobs as motorcycle taxi drivers, hairdressers, or ran small shops. On the compounds children had access to a number of everyday objects such as jerry cans, bowls, plastic crockery, or fabric. Parents’ formal education level was comparatively low: Most fathers and some mothers finished primary school, and very few completed further education. Children’s families spoke Swahili, Alur, or Acholi at home, and often a mixture of these languages. Data were collected in four villages in the Masindi district, Uganda, during February 2013.

Germany: The spontaneously occurring behaviour of six children (two girls and four boys) aged 343–642 days, was observed and recorded during playtime at a daytime nursery, and while seated in a shared trolley on a trip around town. The nursery consisted of one group of 12 children, all of them under 2 years of age. Three staff members took care of and interacted with the children. Nursery staff spoke German with the children, but some children were bilingual, with either one or both parents speaking a different native language. Children’s parents were mainly staff at the local university or university hospital. Parents’ formal education level was high; at least one parent of each child was educated to degree level. The nursery group was housed in a separate building with a small outside play area. Children had access to a group room with a small building area, tables and chairs, and a climbing platform, a long corridor, and a kitchen and bathroom. Children sometimes used the garden with a sandbox, outdoor toys, tricycles, and playhouse. The children often went on trips around town on a specially made trolley. A nursery staff member/carer was always present or nearby. During the free play children were able to move around and interact with the environment (which included building toys, books, stuffed toys, and toy cars), with adults, and with other children of a similar age; during the trolley trip children were more restricted in their movement but could interact with the adults and other children in the trolley. Data were collected in Göttingen, Germany, during August and September 2014.

### Procedure

Data collection was all occurrence sampling (Altmann 1974) with all communication instances produced by the focal child or occurred in the group around the focal individual recorded. All social interactions were recorded using a high-definition camcorder (Panasonic HC-V380) from a variable distance (usually between 1 and 8 meters) depending on the visibility and the children’s movement.

### Analysis

All videos were watched through for potential cases of gestural communication. Signals were coded within a customized sheet on FileMaker Pro Advanced 11.0v4 and data subsequently exported into Excel for Mac (15.19.1). Where necessary, data were converted to means for each individual, to remove any effect of pseudoreplication from the variable amount of data collected per individual. Analyses were carried out in SPSS (v23) with *α* = 0.05 required for significance. Means are given ± Standard Deviation, throughout. Data were examined for appropriateness for parametric statistics and where necessary transformations applied and the data re-tested. Where this was the case the data are clearly marked. Where no appropriate transformations were possible non-parametric alternatives were used. All statistical tests were two-tailed.

In the analysis of our data we chose to focus on gestures alone, and not to analyse any other accompanying signals such as vocalisations or spoken words. A large body of literature suggests infants’ gestures and are often combined with vocal behaviour (e.g. Blake et al. 2005, Acredolo and Goodwyn 1988), and this was also frequently the case in our sample. However, here we limit our analysis to the gestures for a more direct comparison to the gestural behaviour of other apes.

### Identifying gestures

Following the method used in Hobaiter and Byrne (2011a, b), gestures were defined as: ‘discrete, mechanically ineffective physical movements of the body observed during periods of communication’. These movements included movements of the whole body, limbs and head, but not facial expressions or static body postures. So, for example, where a child supported itself by grabbing another’s clothes, or moved a recipient into position by pushing them, these actions were not classed as gestures as they were mechanically effective in achieving their own goal. However, where a child pushed on a recipient and then released them and the recipient subsequently moved into position, this was classified as a potential gesture. This classification may result in the exclusion of some actions that are gestures; however, it prevents the accidental inclusion of actions that are not gestures.

Given evidence for a largely biologically-inherited available repertoire of ape gestures, with greatest overlap between the most closely related species (Byrne et al. 2017; Hobaiter and Byrne 2011a, b; Graham et al. 2017), we initially employed the repertoire used to describe the species most closely related to humans, for which we have the largest data set: chimpanzees (Hobaiter and Byrne 2011a, b, 2017 and in unpublished data) updated in). However, we checked any apparently novel gesture types outside of this against the repertoires described for other great apes (Byrne et al. 2017), which led to the addition of four gesture types from the gorilla repertoire: ‘Hit self’ ‘Object on head’ ‘Rub’ and ‘Tapping self’, and the description of two new (to the nonhuman ape repertoire) gesture types: ‘Arm bend’ and ‘Hand wave’ (see Table 1 for definitions).

**Table 1:** A gestural repertoire of 1- to 2-year-old children

Gesture type (gestural action)	Definition	*N*	Chimpanzee		
			I	J	All
Arm bend^a^	One or both arms held horizontally away from the signaller and then rapidly retracted towards the signaller	10	−	−	−
Arm raise	Raise arm and/or hand vertically in the air	56	+	+	+
Arm raise w. object	As ‘Arm raise’, while holding object	10	−	+	+
Arm shake	Small repeated back and forth motion of the arm, and/or hand	5	+	+	+
Arm wave	Large repeated back and forth motion of the arm raised above the shoulder	7	−	+	+
Arm wave w. object	As ‘Arm wave’, while holding object	5	−	+	+
Bite	Recipient’s body is held between the teeth or lips of the signaller	2	+	+	+
Clap	Both palms moved towards each other and are brought together with audible contact	6	^1^	−	^1^
Embrace	Signaller wraps one or both arm(s) around the recipient and maintains physical contact	3	+	+	+
Fling	Rapid movement of the arm or hand in the direction of the recipient	1	^1^	+	+
Grab	Hand is firmly closed over part of the recipient’s body	16	+	+	+
Grab hold	As ‘Grab’ but closed hand contact is maintained for at least 2 s	11	+	+	+
Grab pull	As ‘Grab’ but closed hand contact is maintained and a force exerted to move the recipient from their current position	8	+	+	+
Grab pull 2hands	As ‘Grab pull’ but with both hands	1	+	+	+
Hand on	Palm of the hand or knuckles are placed on the recipient, contact lasts for more than 2 s	1	+	+	+
Hand wave^a^	Repeated back and forth motion of the hand from the wrist, typically while held above the shoulder	21	−	−	−
Head shake	Repeated back and forth movement of the head (side to side or vertical)	7	−	+	+
Hit object/ground	Movement of the arm from the shoulder with hard short contact of the open palm or closed fist to an object or the ground	10	+	+	+
Hit object/ground 2hands	As ‘Hit object/ground’ but with both hands	2	+	+	+
Hit object w. object	As ‘Hit object/ground’ but the hand holds an object which is brought into contact with another object or the ground	5	−	+	+
Hit other	As ‘Hit object/ground’ but the hand is brought into contact with the recipient’s body	17	+	+	+
Hit other 2hands	As ‘Hit other’ but with both hands	3	+	+	+
Hit self^b^	As ‘Hit other’ but the hand is brought into audible contact with the signaller’s body	10	−	^1^	^1^
Hit with object	As ‘Hit other’ but the hand holds an object which is brought into contact with the recipient’s body	3	+	+	+
Hitting object/ground	As ‘Hit object/ground’ but there is regular rhythmic repetition of the action	8	^1^	^1^	+
Hitting other	As ‘Hit other’ but there is regular rhythmic repetition of the action	5	+	+	+
Hitting with object	As ‘Hit with object’ but there is regular rhythmic repetition of the action	1	−	−	+
Jump	While bipedal both feet leave the ground simultaneously, accompanied by horizontal displacement through the air	2	+	+	+
Locomote	An exaggerated stiff walking or running movement, typically with audible contact of the feet	2	+	+	+
Look	Signaller holds eye-contact position with the recipient for at least 2 s	3	^1^	^1^	+
Object in mouth	Signaller approaches recipient while carrying an object in the mouth	2	+	+	+
Object on head^b^	Signaller faces or approaches recipient while balancing an object on the head	4	−	^1^	^1^
Object move	Object is displaced in one direction, contact is maintained throughout movement	2	+	+	+
Object shake	Repeated back and forth movement of an object	10	+	+	+
Poke	Firm, brief push of one or more fingers into the recipient’s body	1	^1^	+	+
Push	Palm in contact with recipient’s body and force is exerted in attempt to displace recipient	7	+	+	+
Reach palm	Arm extended to the recipient with the palm held vertically or upwards and the fingers in an open position	107	+	+	+
Reach directed	As ‘Reach palm’ but arm is extended towards a third party or object, while audience checking, response waiting, and/or other signals are directed to recipient	164	−	+	+
Rocking	Back and forth movement of the torso from the waist, typically while seated	6	^1^	+	+
Rub^b^	Back and forth movement of the palms on the signaller’s body	32	−	−	−
Stomp	Sole of the foot is lifted vertically and brought into contact with the surface being stood upon	11	+	+	+
Stomping	As ‘Stomp’ but there is regular rhythmic repetition of the action	1	+	+	+
Swing	Large back and forth movement of arm(s) or leg(s) from shoulder or hip	3	+	+	+
Swing w. object	As ‘Swing’ but hand or foot holds an object	7	−	+	+
Tap object	Movement of the arm from the wrist or elbow, with firm short contact of one or more fingers to the object	2	−	−	+
Tap other	As ‘Tap object’ but the fingers are brought into contact with the recipient’s body	3	+	+	+
Tapping object	As ‘Tap object’ but there is regular rhythmic repetition of the action	6	−	^1^	+
Tapping other	As ‘Tap other’ but there is regular rhythmic repetition of the action	6	−	+	+
Tapping self^b^	As ‘Tapping other’ but the fingers are brought into contact with the signaller’s body	3	−	−	−
Throw object	Object is moved and released so that there is displacement through the air after moment of release	36	+	+	+
Thrust	Hips are brought into repeated contact with the recipient’s body	1	−	−	+
Touch	Light contact of the hand and/or fingers on the body of the recipient, contact under 2 s	25	+	+	+

Within the chimpanzee repertoire we distinguish those recorded to date as employed by infant (I) or juvenile (J) chimpanzees, from those recorded in the species repertoire (All); + = present, − = not observed; ^1^observed but insufficient cases in wild chimpanzees for inclusion in current repertoire. Gesture descriptions follow: Hobaiter and Byrne 2011, updated in Hobaiter and Byrne 2017. *N* = number of gesture tokens. Given a small data set, all potential gestures are described here, however we would typically require *n* = 2 cases of intentional use and use by at least two individuals for inclusion in a great ape repertoire (Hobaiter and Byrne 2011a, b)

^a^The gestures have not been described to date in the repertoire of other apes

^b^The gesture is found in the gorilla, but not in the chimpanzee repertoire; all other gestures are present in the chimpanzee repertoire

Intentional communication was behaviourally defined as per Hobaiter and Byrne 2011a, b, and is coherent with the definitions across the great ape gestural communication literature (including for example: Tomasello et al. 1985; Pika et al. 2005; Liebal et al. 2006; Tanner and Byrne 1993; Genty et al. 2009; Roberts et al. 2012; Frohlich et al. 2016). We considered an action of the body to be an intentional gesture only where it was targeted to a particular recipient with the aim of influencing their behaviour in a specific way. We employed Audience checking, Response waiting, and Persistence, as behavioural indications of intentional usage (see Hobaiter and Byrne 2011a, b for detailed definitions for this coding scheme), and required each potential case of gestural communication to be accompanied by one or more of these to be included as an intentional gesture in analyses. In our non-human great ape research potential cases of gesture apparently directed towards the researcher holding the camera are excluded because of the species difference between researchers and subjects. However, given that this was not the case in this study, where the appropriate criteria were met, we included them in our gestural coding.

### Structure of gestural communication

As seen in great ape gesturing, the production of gestures and other signals by children were not necessarily a clear production of a single signal followed by a response. Instead gestures, and other signals, could be produced in series, could overlap with each other, and could be exchanged. As in chimpanzee gesturing (Hobaiter and Byrne 2011a, b) we distinguished three discrete structural phases in child gesturing. Where a gesture or several gestures occurred within a pause of 1 s or less between gestures they were coded as part of the same *sequence*. If more than one sequence with a pause of more than 1 s between them occurred, they were categorized as part of the same *bout*. Whenever gestures were exchanged back and forth at least once between the signaller(s) and the recipient(s) we categorized them as an *exchange*. Rather than imposing a time limit within which a behaviour was considered to be given as a response to a prior communication, we took intermediate behaviour directed towards the recipient (for example: audience checking, or holding the position of a body part presented) and the absence of either non-directed behaviour (such as self-grooming, or feeding), or behaviour directed towards another individual, to indicate that response waiting, and, therefore, communication, was on-going.

As gesturing took place in the children’s natural environment multiple individuals may be present while they were signalling. We coded signals as belonging to one of four possible recipient contexts: (1) One certain recipient: a single potential recipient is present, the signaller makes contact with them during a gestural action (e.g. a Touch gesture), and/or directs eye-gaze during audience checking (prior to or during signalling) or response waiting (after or between signalling) towards them. (2) Several recipients but directed to one individual: several potential recipients are present, but the signaller directs communication to one individual as indicated by contact during a gestural action or ye-gaze as before. (3) One potential recipient: only one individual is present; but no indication that signalling is directed to that individual. (4) Several potential (several potential recipients but with no indication that behaviour is directed to any one individual). Only actions coded with recipient as either ‘One certain recipient’ or ‘Several recipients but directed to one individual’ were considered to be cases of intentional communication and used in analyses.

In addition, each individual gesture was coded for Modality (Silent-visual, Audible, Contact); Situational context of the signaller immediately prior to signalling (Affiliating, Bathing, Feeding, Grooming, Nursing, Play social, Play solitary (with others present), Traveling, Unknown); and Recipient’s response (Gesture, Other action, or None). In comparisons with the chimpanzee data, early coding of chimpanzee gesture incorporated the context of the communication, rather than the context immediately prior to it (e.g. in Hobaiter and Byrne 2011a, b). As a result, the social use of gestures immediately following solitary play in order to engage an adult in social play would have been coded as ‘Social play’ in earlier data. To address this discrepancy we provide the data on solitary and social play both separately and in combination (‘Play’) here. We classified gestures produced whenever children were not able to move freely, for example when bathing or while sitting in the travelling trolley as ‘Restricted’ with all other gestures classified as ‘Free’.

### Identifying goals and meaning in gestural communication

As in ape gesturing (Cartmill and Byrne 2010; Hobaiter and Byrne 2014), we defined the goal as ‘the behavioural response of the recipient that satisfied the signaller, as indicated by the signaller stopping signalling’. Goals were established based on the behaviour observed in the human infants; however, as these reflected goals previously established in the ape literature (e.g. Genty et al. 2009; Hobaiter and Byrne 2014) we employed the same labels for convenient comparison. As a result only successful gestures can be assigned a goal. We assigned gestures to the following goals: Acquire object, Affiliation, Direct attention, Follow me, Move away, Move closer, Pick me up, Stop behaviour, Travel with you, and Play. We investigate the goals of gestures both with and without play data, as play as a behavioural context may incorporate the playful use of ‘real-world’ goals such as ‘Move away’ or ‘Move closer’ (Hobaiter and Byrne 2014). We employ the term meaning to refer to the consistent use of a gesture across multiple instances to achieve the same goal either by one individual (idiosyncratic meaning) or across individuals. Gestures may be tightly associated with a single “meaning”, or flexibly with several (Cartmill and Byrne 2010; Hobaiter and Byrne 2014).

### Definition of potentially referential gestures

We defined gestures as ‘directed’ where they appeared to direct the recipient’s behavioural response in a particular direction. For example: *Directed push*, the recipient moves their body into the location indicated by the push action. Please note that all gestures are directed in the sense of being goal-directed, and directed towards a specific recipient; here we specify those that appear to be referential, in that they are directed to an external location or third party. We distinguished a ‘Reach directed’ gesture from a typical ‘Reach’ gesture following Hobaiter et al. (2013) as: a ‘Reach’ gesture that was oriented towards a third party or object that was spatially distinct from the recipient (in the case of direction towards a third party, the recipient was identified from the direction of gaze during Audience checking and Response Waiting). In the children we further distinguished the sub-category ‘index-finger point’ (‘Reach directed’ gesture produced with the index finger only extended) from the ‘whole-hand’ (‘Reach directed’ gesture produced with all fingers extended) based on hand shape rather than function. Both of these forms would have been categorized as forms of the single ‘Reach directed’ gesture type were they to occur in another ape species. As a result we do not distinguish them as separate gesture types in comparison of the gesture repertoire. However, as they are frequently considered separately in the human infant literature (e.g. Cochet and Vauclair 2010; Liszkowski 2008; Blake 2000; Carpenter et al. 1998; Tomasello and Camaioni 1997; Franco and Butterworth 1996; Blake et al. 1994; Leung and Rheingold 1981; Acredolo and Goodwyn 1988; Bates et al. 1975) we do discuss the relative frequency of the two forms.

### Chimpanzee data

We compare our findings with data from our work on chimpanzee gestural communication (Hobaiter 2010; Hobaiter and Byrne 2011a, b, 2012, 2014, 2017; Hobaiter et al. 2013, 2017; Hobaiter, unpublished data). We have indicated samples sizes for individual tests in the “Results” section; however, here we provide a summary of the dataset. Data used in these analyses were collected between 2007 and 2017. Chimpanzee signallers ranged in age from 12 months old to approximately 51 years old, and data were collected across all behavioural contexts. The available data set contained 5908 tokens of intentional gesture use: *n* = 782 (13%) from infant chimpanzees under 5 years old (note that no intentional gesture use was recorded in chimpanzees < 1 year old), and *n* = 2129 (*n* = 36%) from juvenile chimpanzees aged 5- to 10 years old. As, in some cases, we compared our data with specific previously published data sets, where there was variation from these numbers they are reported together with the test.

### Inter-observer reliability

AS coded the child videos. CH, an experienced coder of chimpanzee gestural communication, provided training using chimpanzee gestural videos. CH conducted inter-observer reliability testing of 100 cases of potential gesture use by the children, approximately 13% of the dataset (*n* = 788 cases). We employed a random number generator to assign all individual gesture cases a random number between 0 and 1. Cases were then sorted on these random numbers from low to high and the first 50 cases selected for each cohort (Ugandan and German) with inter-observer reliability testing on three aspects: Is the gesture directed to another individual? What is the attentional state of the recipient? And, what is the gesture type? (as per Hobaiter and Byrne 2011a, b). Percentage agreements were high (directedness = 84%; attentional state of recipient = 70%; gesture type 92%), and a good to very good level of agreement was achieved on all three variables (Cohen’s kappa: directedness *K* = 0.700; attentional state of recipient *K* = 0.601; gesture type *K* = 0.841).

### Ethical statement

Parents volunteered for their children to take part in the study. Participation was entirely voluntary with no financial incentives. Participants were informed about the aims of the study and what participation would entail. All parents gave their written consent for their children to take part in the study. For the Ugandan participants, all consent and debriefing forms were translated into Swahili and, if necessary, read and explained with the help of a local field assistant. After completion, participants were debriefed about the nature of the study. The study was performed in accordance with the ethical standards of the University of Göttingen, the Max-Planck-Institute for Psycholinguistics, the University of St Andrews Teaching and Research Ethics Committee, the Ugandan National Council for Science and Technology, and with the 1964 Helsinki declaration and its later amendments.

## Results

We recorded 925 min of communication from 13 children aged 315–642 days (approx. 11–21 months). Ugandan children: total *n* = 493 min: BU: F, 315 days, 60 min; DC: F, 384 days, 61 min; FG: F, 408 days, 63 min; MK: F, 316 days, 96 min; MU: M, 344 days, 72 min; MA: F, 421 days; and P: M, 421 days, 72 min. German children: total *n* = 432 min: BM, M, 633 days, 90 min; FJ: F, 343 days, 27 min; JF: F, 554 days, 72 min; JS, M, 642 days, 108 min; MQ: M, 614 days, 105 min; TD, M, 360 days, 30 min.

### Repertoire

This produced a data set of 788 potential gestural actions (Ugandan children *n* = 390; German children *n* = 398); classified into 52 potential gesture types (see Table 1). 680 of these gestural actions met the strict criteria for definition as a case of intentional gesture typically applied to ape communication. These included all 52 gesture types (see Table 1); only these intentional cases of gesturing are used in the analyses below. We would typically require *n* = 2 cases of intentional use and use by at least 2 individuals for inclusion in a great ape repertoire (Hobaiter and Byrne 2011a, b); however, given a small data set, all gestures are presented here together with the number of cases observed.

**Fig. 1:**
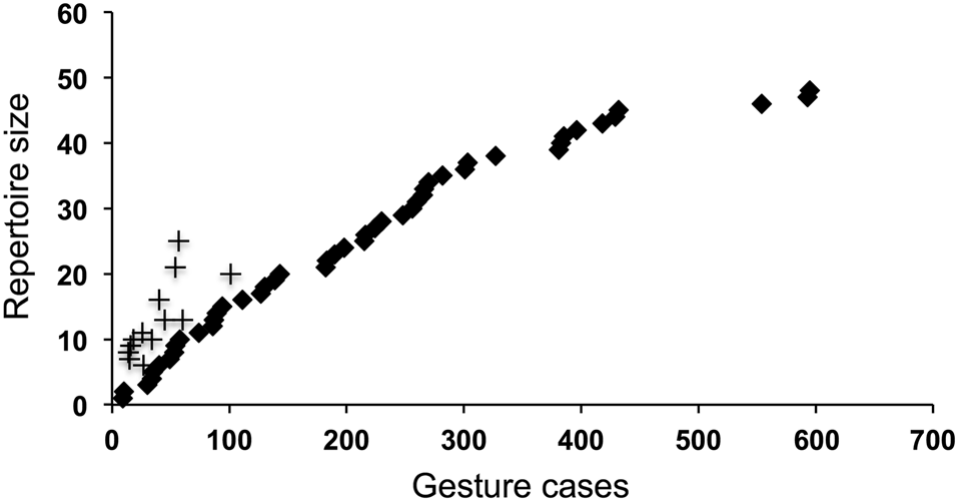
Cumulative record of gesture types in 1- to 2-year-old children. The cumulative number of gesture types is plotted against the number of gesture cases coded (solid diamonds). The total repertoire appears to be starting to approach, but has not yet reached, asymptote. In addition, on the same axes, individual repertoire sizes are plotted against total number of gesture cases (crosses). No individual repertoire approached asymptote

Examining the cumulative frequency of gesture types, as used by any child, suggests that the combined repertoire across children had only started to approach asymptote, and other gesture types remain very likely to be identified (Fig. 1). No individual repertoire approached asymptote, and mean individual repertoires varied considerably (*n* = 13, range 6–28, mean = 15.6 ± 6.5), but correlated closely with the number of gestures recorded per individual (*n* = 13, gestures recorded per individual: range = 22–124, mean = 52.3 ± 33.4; Pearson’s correlation, *r* = 0.75, *n* = 13, P = 0.002; see Fig. 1), suggesting that individual repertoire size in our data was limited by the size of the data set. *N* = 11 gestures were employed by a single child, and could, therefore, be described as idiosyncratic. However, 7 of these were only coded as used in intentional communication on a single occasion, and the other 4 on *n* = 2 occasions. The single use cases would not normally be included in a gestural repertoire, see above, but are included here for completeness given the small data set. Given both the correlation between frequency of gesturing and number of gesture types used described above, and the correlation between the number of times a gesture occurred in a child’s repertoire and its frequency in the data set (Pearson’s correlation, *r* = 0.80, *n* = 51, *P* < 0.0001), the individual repertoires and the frequency with which a gesture was used by different children appear to be determinedly largely by sample size in our data.

### Does the gestural repertoire for 1- to 2-year-old children match that described for other apes?

46 of the 52 gestures described for children were also present in the chimpanzee repertoire, an overlap of 89%. Of the six remaining, four of the gestures used by children: ‘Hit self’, ‘Object on head’, ‘Rub’ and ‘Tapping self’, are present in the gorilla repertoire, and two gestures: ‘Arm bend’ and ‘Hand wave’, were specific to children (see Table 1). ‘Reach directed’ (*n* = 164) and ‘Reach palm’ (*n* = 107) were the most common gesture types observed in children and included almost twice as many cases as the next most typically used gestures (‘Arm raise’ *n* = 56 and ‘Throw object’ *n* = 36, see Table 1). Our data set of infant chimpanzee gestures (*n* = 782 gesture tokens) is, like our child data set, relatively small and we caution against absence in our data representing genuine absence; however, here 28 of the 52 (54%) gesture types used by children were present. The overlap increasing to 39 gesture types (75%) when compared with juvenile chimpanzees, for whom we have a larger data set (*n* = 2129 gesture tokens).

### Context

In young chimpanzees social play was the primary context recorded prior to a signal being produced (infant chimpanzees: 644/782, 82%; juvenile chimpanzees: 1500/2129, 74%); once mature, use in play decreased and gestures were recorded with consortship (440/2997, 15%), grooming (489/2997, 16%) and, travelling (353/2997, 12%), with a similar frequency to social play (487/2997, 16%) in adult chimpanzees. In human children social play (199/680, 29%) and travelling (221/680, 33%) were the most common contexts recorded prior to signalling. Intentional gesture use was also recorded from signallers who had been in a context of solitary play (108/680, 16%), feeding (51/680, 8%), bathing (14/680, 2%), affiliation (5/680, 1%), and nursing (1/680, 0.1%), as well as following unknown contexts (74/680, 11%). Given minor variation in the classification of behavioural contexts over the longitudinal chimpanzee dataset, combining social and solitary play in the child dataset provides more appropriate comparison with chimpanzee data (307/680, 45%).

### The structure of gestural communication in 1- to 2-year-old children

Children employed the majority of their gestures singly (*n* = 449/680, 66%) but produced *n* = 99 gesture sequences containing *n* = 231 gestures. The majority of gesture sequences were produced as gesture pairs (2-gesture sequences, *n* = 72/150, 73%), but they used sequences of up to 5 gestures without pause for response waiting. They showed persistence where a single gesture or gesture sequence failed, producing bouts of 2–7 individual gestures or gesture sequences interspersed with response waiting (2-sequence bouts, *n* = 85, 3–9 sequence bouts, *n* = 46).

### Onset of gesture types and modalities in 1- to 2-year-old children and other apes

The gestural repertoire for children included all three modalities of gesture: Silent-visual *n* = 22 (e.g. ‘Arm raise’, ‘Hand wave’), Contact *n* = 18 (e.g. ‘Grab-pull’, ‘Poke’), and Audible *n* = 12 (e.g. ‘Hit object/ground’, ‘Clap’). Silent-visual gestures made up 42% of gesture types (22/52), but a mean 71 ± 11% of gesture use across children (*n* = 13); the three most common gesture types (as above, ‘Reach’, ‘Reach-directed’, and ‘Arm raise’) were all silent-visual and together account for almost half of all gesture use (*n* = 327/680, 48%). Contact gestures made up 35% of gesture types and 19 ± 10% of gesture use, and Audible gestures made up 23% of gesture types but only 10 ± 8% of gesture use.

In wild chimpanzees (gesture cases *n* = 4397), Silent-visual gestures made up 34% of gesture types and 42 ± 27% of gesture use across signallers (*n* = 69; age 1–51 years); Contact gestures made up 37% of gesture types and 25 ± 19% of gesture use, and Audible gestures made up 29% of gesture types and 34 ± 26% of gesture use (Hobaiter 2010).

**Fig. 2:**
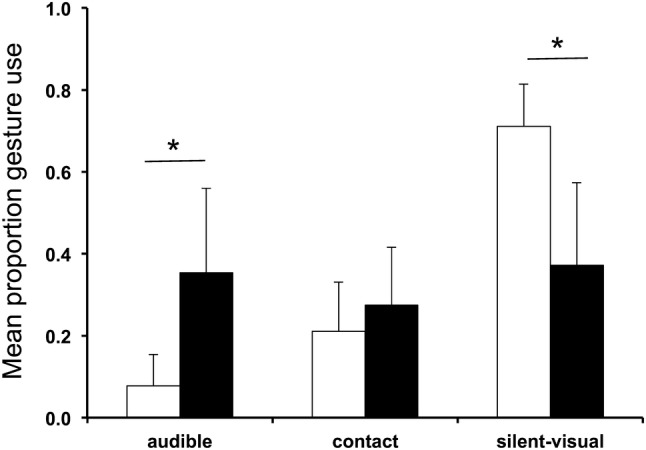
Comparison of the mean proportion of gesture use across modalities (audible, contact, and silent-visual) by chimpanzees and children. White bars represent children; black bars represent chimpanzees; error bars represent standard deviation. Planned *t* tests were used to explore differences between the use of modalities in the two species: *P < 0.0001

As expected, given the large overlap in repertoires, the distribution of modality of gesture types did not vary between chimpanzees and 1–2 year old children (Chi square χ^*2*^ = 1.91, *df* = 2, *P* = 0.38); however, the frequency of use of gesture modalities did in the use of both audible and silent-visual, but not contact gestures (*n* = 13 children; *n* = 50 chimpanzees: ANOVA *F* = 16.74, *df* = 2, *p* < 0.001. Planned post-hoc *t* tests; audible: *t* test_equal variences not assumed_*t* = − 7.03, *df* = 51.9, *p* < 0.001; contact: *t* test *t* = − 1.95, *df* = 61, *p* = 0.056; silent-visual: *t* test_equal variences not assumed_*t* = 8.32, *df* = 36.49, *p* < 0.001. See Fig. 2).

### Do 1- to 2-year-old children’s gestures have flexible meanings?

We had insufficient cases of successful use per individual for each gesture type to explore flexible use of a specific gesture towards a particular goal. As a result, we had to combine data across individuals. While this has been done in the ape literature where it has been shown that individual identity did not impact signal meaning (e.g. Hobaiter and Byrne 2014; Graham et al. 2017), we are cautious in our interpretation of the following analysis as we are unable to control for the impact of individual identity.

Thirty-nine gesture types were used to successfully achieve at least one of ten goals. Nine of these were imperative: Acquire object, Affiliation, Follow me, Move away, Move closer, Pick me up, Play with me, Stop that, Travel with you, and one potentially declarative: Direct attention. 16 gesture types were used successfully on 3 or more occasions (14 of these 16 were used by more than one child successfully, range 1–12 signallers per gesture type, mean = 4.4 ± 3.2). Gestures were used to achieve a mean 3.1 ± 1.3 goals (range 1–6 successful goals per gesture); however, 14 of the 16 gestures were used towards a play goal. If we consider only non-play goals gestures were used to achieve a mean 2.3 ± 1.3 goals (range 1–5 successful goals per gesture), suggesting that individual gestures were used flexibly to achieve different goals.

### Referential communication

We observed referential *n* = 164 ‘Reach directed’ gestures from all 13 children, including the two youngest individuals (315, 316 days). Within the ‘Reach directed’ gestures the index-finger point form (*n* = 119) was far more common than the whole-hand reach form (*n* = 45; Binomial test *P* < 0.0001). Referential use of gestures was far more frequently observed in humans than in a similar study of wild chimpanzees that employed the same criterion (oriented to a third party or object spatially distinct from the recipient, see Methods: children *n* = 164/680; chimpanzees *n* = 4/4397; Fisher’s exact test *P* < 0.0001; chimpanzee data from Hobaiter et al. 2013, *n* = 2 juvenile chimpanzee signallers, age = 5-years in both cases).

## Discussion

Using an ethological approach developed for the study of great ape gestures, we found that 1- to 2-year-old children employed a large repertoire of 52 gesture types in intentional contexts, as defined by objective behavioural criteria (following: Bates et al. 1975; Tomasello et al. 1985; Genty et al. 2009; Cartmill and Byrne 2010; Hobaiter and Byrne 2011a, b). Only two gesture types were specific to human children in our sample: Arm bend and Hand wave. In addition, within the Reach directed gesture type, the majority (73%) of cases were of the index-finger pointing form (as compared to the whole-hand reach form), which has not been recorded in the repertoire of wild apes (Hobaiter et al. 2013; Byrne et al. 2017). In contrast 50 gestures (96%) were shared between children and other apes in our data set, and 46 gestures (89%) between children and chimpanzees.

This paper is the first to attempt to compare the spontaneous gesture repertoire of human infants to those of other apes in their natural habitat using a methodological approach that is directly comparable. Our results report both the types of gestures and the frequency in which we observed them in everyday interactions. Our subjects were human infants in their second year of life observed in natural habitat situations: at home and at a daytime nursery. While our data represent a demonstration of the use of gestures from the ape-typical repertoire in communication by 1- to 2-year-old children, our sample size remains small both in terms of the number of individuals and the number of gesture instances, with some gesture types observed on only a single occasion. As a result, we are cautious about our interpretation of our findings. One criticism of comparative psychology has been the tendency to compare the behaviour of young children, with that of nonhuman apes of all ages (Leavens et al. 2017). Here we have provided an indication of where the gesturing of infant, juvenile, and mature chimpanzees varies. We note, for example, that children’s use of gesture following both travelling and play seems more similar to the use of gesture in adult chimpanzees. However, the use of all occurrence sampling prevents a direct comparison of rates of gesturing across contexts.

Exploring communication within the full range of contexts that require its expression is key to our understanding of both a repertoire and its use (Hobaiter and Byrne 2011a, b; Seyfarth and Cheney 2017). Given the sample size and variation in the behavioural contexts recorded between the two populations, we are limited in our ability to explore any potential cultural differences. However, while we are unable to explore the absence of gesture types, of those gesture types recorded on more than 5 occasions in human children, 24 (80%) were present in both human and chimpanzee populations, adding weight to the argument that many of the gestures we documented may be part of a universal repertoire of available gestures, shared across human and nonhuman ape species.

The presence of a universal repertoire may indicate that these gestures are ‘innate’ in the traditional sense of the term (i.e., morphogenetically predetermined as part of the behavioural repertoire of all apes, the products of innate blueprints). However, they may also be conceived of as the emerging final products of evolutionary and developmental constraints in ontogeny (see Bertossa 2011, for an evolutionary-developmental approach to morphology and behaviour). For example, ‘Hit Object’ or “Tap object” may be the final product of the interaction between flexible manual action patterns common to all ape species (themselves an emerging evolutionary-developmental feature of their bodies and basic action patterns) and the constraints encountered in the dynamics of social interaction, where they are discovered as useful actions to regulate basic interaction patterns shared across ape species. However, only a fraction of the manual action patterns possible are employed as intentional gestures (Hobaiter and Byrne 2017). Irrespective of the origin of available gesture types in the repertoire (the ‘tool kit’), their use in day-to-day gesturing (the ‘tool use’)—the choice of gesture type and the social and physical nuances of how it is deployed in a specific interaction—may be highly flexible (Bard et al. 2017; Fröhlich et al. 2017; Fröhlich and Hobaiter 2018; Hobaiter and Byrne 2011; Liebal et al. 2014, 2018; Pika and Fröhlich 2018).

Children’s use of gestural sequences was also similar to other apes. The majority of gestures were produced singly (66% in children; 61% in chimpanzees, Hobaiter and Byrne 2011). As in other apes (Liebal et al. 2004; Genty and Byrne 2010; Hobaiter and Byrne 2011), humans produced gestures both in rapid sequence without pause for response waiting, as well as in bouts of gesturing interspersed with response waiting when initial gestures failed. Finally, humans, like chimpanzees (Hobaiter and Byrne 2011), produced the majority of their gesture sequences as gesture pairs.

Children’s use of the different modalities of gesture available may also differ slightly from that of chimpanzees’. Unsurprisingly, given the apparently large overlap in repertoire, the distribution of silent-visual, audible, and contact gesture types within the repertoire was very similar. However, selective use of the gestures within the repertoire appeared to vary. So that while both children and chimpanzees employed silent-visual gestures more often that other modalities, the skew towards use of silent-visual gesture use appears stronger in children. The use of signals from within the species repertoire varies across behavioural contexts in chimpanzee communication (e.g. Tomasello et al. 1985; Genty et al. 2009; Hobaiter and Byrne 2011a, b; Hobaiter et al. 2017), as does vocabulary in human language. In addition, primate signalling varies with changes in the physical environment (Brown and Wasser 1988), which impact the transmission of visual and acoustic information. As a result, this skew may reflect a genuine species difference between human and chimpanzee gesturing; or it may reflect variation in the behavioural context, or in the visual and acoustic environment in which gesturing occurred.

In this study, we focus on the analysis of gestural communication to provide a direct comparison with the work done on other great ape species. While we did not analyse vocalisations or words that accompanied gestures, children in our sample were at the cusp of spoken language and did combine both vocalizations and words with their gestures. The use of spoken language represents a challenge for direct comparison with non-human ape communication; however, with a wider age-range, it may be productive to explore how the use of the gestural repertoire changes with the onset and development of spoken or signed language.

This apparently substantial overlap in the repertoire and use of gestures we observed in our sample suggests that before or at the early onset of language proper, human infants’ gestural repertoire is, at some level, largely shared with other apes, and they display it in a similar fashion: with indications of intentional use, in combination with different gestures, and flexibly towards more than one specific goal. We have come full circle: the intentionality criterion used to first describe the non-verbal intentional behaviour of young human children (Bates et al. 1975), provided the method on which the study of intentional signal use in non-human apes was based, and now allows for systematic comparison of intentional gesture use across human and non-human ape species. We suggest that these gestures have a long evolutionary history and may continue to be present in older language users, still existing alongside the other gestures that accompany speech or conventional gestures learned in a cultural context (for example: waving to say good-bye, the ‘thumbs-up’ gesture, or culturally specific forms of pointing).

Apes perform a number of gestures that involve touching or manipulating objects or even a recipient’s body, for example drumming or slapping the ground (Pika et al. 2003; Liebal et al. 2007; Hobaiter and Byrne 2011a, b). Traditionally these kinds of gestures have not been included in descriptions of infant gesturing, being instead classified as functional object use or “meaningful action” (e.g. Acredolo and Goodwyn 1988; Iverson and Goldin-Meadow 2005). As Iverson et al. (1994) and Capirci et al. (2005) suggest, the exclusion of these gesture types likely leads to an underestimation of the gestural repertoires of human infants (see also Gómez 2015). In this study we show that infants indeed produce a variety of gestures involving objects and recipients in a way that meets behavioural criteria for intentional use, and that these should be included in their gestural repertoire.

As in human language, some great ape gestures are associated with a single meaning, while others are employed flexibly to achieve several (I put my money in the *bank*; I walked along the river *bank* to my boat; Cartmill and Byrne 2010; Hobaiter and Byrne 2014). Children, like other great apes, appear to use their gestures to consistently achieve particular goals, with some gestures associated with a single goal and others with several. The description of specific meanings with individual gesture types requires a substantial dataset; thus, it remains possible that those gestures employed by children in this data set to achieve a single goal might also be used flexibly towards others. Children in our sample used their gestures flexibly to achieve around two goals per gesture type, after play data were excluded. These data are remarkably similar to those found in chimpanzees who employed their gestures towards an average of three goals, but with one typically used to achieve play (Hobaiter and Byrne 2014). Similar flexibility has been observed in other studies of specific human gestures—for example index finger pointing has been observed to achieve a wide variety of goals: requesting an object, expressing and aligning attitudes about objects and event, or helping a receiver by providing information (Liszkowski et al. 2005a, b), and perhaps (in an interrogative way) requesting more information about object properties (Begus and Southgate 2012; Southgate et al. 2007).

Our ethological approach focuses on the form and function of gestures, and their intentional use in ecologically valid contexts. This approach allows us to consider how gestures are used in the child’s everyday interactions, their intentionality, and the full repertoire of gestures rather than specific types, such as deictic gestures. While only involving two populations, our study shows the feasibility and significance of this ethological approach. To investigate the infant’s full gestural repertoire and its combination with word or sign as language comes online, needs a far larger data set incorporating more individuals as well as a wider range of communicative contexts and cultural variation.

It has been proposed that one major difference between primate and infant gestures is that other apes mainly produce imperative gestures whereas human infants produce many, or even mostly, protodeclarative and informative gestures (Gómez et al. 1993; Tomasello and Camaioni 1997, Tomasello and Herrmann 2010; Tomasello 2008). There has been a tendency in comparative research to highlight these supposedly uniquely human protodeclarative gestures as the most relevant form of communication in infants, which might be indicative of an important divergence in the motivational and cognitive mechanisms controlling gestural communication in humans. Our results suggest that human infants do not engage preferentially in declarative communication and that the full range of gesture types should be considered in comparative work. Nevertheless, our sample may not include contexts in which declarative gestures are much more common, for example one-on-one time with a caregiver, a setting which would invite joint attentional episodes and exploring the environment together (Liszkowski and Tomasello 2011; Liszkowski et al. 2012). It is also possible that there are significant cultural differences in the frequency of joint attentional episodes and deictic gestures produced by and for the child in our two populations. Salomo and Liszkowski (2013) documented these differences in three different cultures. To the best of our knowledge this is one of the only studies that show how specific forms of interaction and the infant’s everyday activities influence the frequency and repertoire of their gestures—a much needed supplement to studies that are more typically conducted in a lab setting.

Whereas our sample of 1- to 2-year-old infants, on the cusp of acquiring spoken language, represent an ideal cohort for the study of gesture, the absence of any asymptote in the repertoire means that some gesture types in the human repertoire have been missed. Future research should extend observations to a wider range of individuals and consider the understanding of gestures, as well as their production. This technique was recently successfully employed to more fully describe the gestural repertoires of wild bonobos (Graham et al. 2017), where, for example, an infant showed understanding of a gesture directed to them by an adult that was not yet expressed in their own repertoire (e.g. to indicate the adult’s goal ‘Climb on me!’).

In sum, we have reported initial evidence that our proposed ethological methodology reveals a complex repertoire of gestures in human infants that has been frequently either neglected or reported in an asystematic way. We observed in our sample that this repertoire largely overlaps with that described in nonhuman apes using the same methods and is employed in a similar way. We suggest that this methodology could be fruitfully employed with larger samples of children and populations to investigate the natural gestural repertoire of human children and other apes from a comparative perspective.
